# Afterdischarges in Myasthenia Gravis

**DOI:** 10.3389/fneur.2021.599744

**Published:** 2021-04-13

**Authors:** Li Yang, Shougang Guo, Xiuying Chen

**Affiliations:** ^1^Electromyography Room, Shandong Provincial Hospital Affiliated to Shandong First Medical University, Jinan, China; ^2^Department of Neurology, Shandong Provincial Hospital Affiliated to Shandong First Medical University, Jinan, China

**Keywords:** peripheral nerve hyperexcitability, MuSK, acetylcholine receptor, Myasthenia Gravis, afterdischarge, repetitive nerve stimulation

## Abstract

**Introduction:** This study aimed to analyze the clinical features of myasthenia gravis (MG) in combination with the afterdischarges and compare the characteristics of afterdischarges in MG with different serum antibodies.

**Methods:** Ninety-two patients with MG were analyzed retrospectively. The afterdischarges were investigated using motor nerve conduction examination, F-wave examination, and repetitive nerve stimulation (RNS).

**Results:** Afterdischarges were observed after the M wave in 14 of 92 patients. Three of these 14 patients tested positive for the muscle-specific tyrosine kinase antibody (MuSK-Ab), and 11 patients tested positive for the acetylcholine receptor antibody (AchR-Ab). The characteristics of the afterdischarges on RNS differed distinctly between the two antibody groups. The afterdischarges occurred on the first stimulation, but decreased on the second and subsequent stimulations in patients with MuSK-MG, while the afterdischarges continued to occur on each stimulation in patients with AchR-MG.

**Discussion:** The characteristics of the afterdischarges on RNS enabled easy identification of their synaptic or neurogenic nature.

## Introduction

Myasthenia gravis (MG) is an immune-mediated postsynaptic membrane disease, which is characterized by fluctuating ocular, pharyngeal, or limb muscle weakness. Studies have found that MG can be associated with peripheral nerve hyperexcitability (PNH), which may in turn be related to the presence of tumors (1–3) administration of acetylcholinesterase inhibitors (AchEIs) (4), or an unclear etiology (5, 6). The presence of afterdischarges after motor nerve stimulation, which may occur prior to the onset of clinical symptoms, is a common feature of patients with PNH (7). In this study, the clinical and electrophysiological characteristics of MG with afterdischarges were analyzed retrospectively, along with a preliminary investigation of the underlying pathological mechanisms of the afterdischarges. Previous studies have reported on MG associated with PNH. However, to the best of our knowledge, this is the first study to compare the characteristics of the afterdischarges of patients with serum muscle-specific tyrosine kinase (MuSK) antibody-positive MG (MuSK-MG) and those with acetylcholine receptor (AchR) antibody-positive MG (AchR-MG).

## Methods

The study design was approved by the Biomedical Research Ethical committee of Shandong Provincial Hospital (No. SWYX2020-026). The committee waived the need for informed consent as there was no direct contact with the eligible participants or any adverse effects associated with the experimental procedure.

### Patients

The data of 104 patients with a confirmed diagnosis of MG who were hospitalized for the determination of the definitive diagnosis or for further treatment at the Department of Neurology of Shandong Provincial Hospital between September 2016 and June 2020 were reviewed in this study. Patients who did not undergo electrodiagnostic (EDX) examination at the hospital were excluded. Consequently, 92 patients were included in the analysis.

Forty-three of the 92 patients were men and forty-nine were women, with a mean age of 53 years (range, 17–77 years).

The diagnostic criteria for MG included typical fluctuating skeletal muscle weakness, positive neostigmine test, and/or abnormal decrement on low-frequency repetitive nerve stimulation (RNS). None of the patients had a history of radiotherapy, toxic exposure, or genetic disorders of the neuromuscular system.

The data of every patient were obtained and analyzed, including the Myasthenia Gravis Foundation of America (MGFA) clinical classification, Quantitative Myasthenia Gravis Score (QMS), thymus computed tomography scans, presence/absence of serum AchR antibody (AchR-Ab)/MuSK antibody (MuSK-Ab), administration of AchEIs, and results of the EDX studies.

### EDX Studies

All patients underwent EDX examination at the hospital. Afterdischarges were investigated using motor nerve conduction, F-wave, and RNS examination. The EDX readers were blinded to the MuSK vs. AchR status. Afterdischarges were defined as repetitive low-amplitude compound muscle action potentials (CMAPs) that closely followed the main CMAP after a single shock. The amplitude and duration of the main CMAPs were usually normal.

In our laboratory, EDX examination for patients with MG routinely includes the following items. At least one side of the median, ulnar, tibial, and peroneal nerves was examined to assess motor nerve conduction in each patient. At least one side of the facial, accessory, and median nerves was examined to assess RNS in each patient. Needle electromyography was performed on at least one side of the tibial anterior muscle, gastrocnemius, first dorsal interosseous muscle, and biceps brachii to identify the presence of spontaneous motor unit firing. F-wave examination was performed in 33 patients in this study.

## Results

### General Characteristics

Sixty-three patients tested positive for the AchR-Ab, while 9 patients tested positive for the MuSK-Ab; 2 patients were double-positive and 10 patients were double-negative. Eight patients did not undergo antibody testing. Twenty-one (32%) of the 65 patients who tested positive for AchR-Abs had thymic abnormalities (thymic hyperplasia or thymoma), 5 of whom had undergone thymectomy.

### EDX Studies

Fourteen of 92 patients presented with afterdischarges after the M wave, with an occurrence rate of ~15%. The afterdischarges were observed at an increment of 5 mV/D in 10 patients, and in the remaining 4 patients when the increment was adjusted to 2 mV/D. Afterdischarges were observed on the tibial nerves, peroneal nerves, median nerves, and ulnar nerve of 10, 6, and 5 patients and 1 patient, respectively. Afterdischarges were not observed on the facial and accessory nerves. Fasciculation and myokymic or neuromyotonic potentials were not observed on needle electromyography, except for patient 1 in whom fasciculation potentials were found in the abductor pollicis brevis and gastrocnemius.

The clinical characteristics of 14 patients with MG with afterdischarges are shown in Table 1. The QMS and disease duration each varied greatly. Five patients were classified as type IIa, 5 patients as type IIb, 3 patients as type IIIb, and 1 patient as type IVb, according to the MGFA classification. Afterdischarges were not observed in patients with type I MG in this study cohort.

**Table 1:** Clinical characteristics of patients with MG with afterdischarges.

**Patient**	**Sex/Age (years)**	**MGFA type**	**QMS**	**Disease duration (months)**	**PB (mg/d)**	**Thymus**	**MuSK-Ab/AChR-Ab**	**Nerves with AD**
1	F/60	IIIb	18	168	400	Normal	+/–	T, P, Me, U
2	F/41	IIb	10	9	180	Normal	+/–	T
3	F/38	IIIb	20	24	180	Normal	+/–	T
4	F/67	IIb	12	2	N	Thymoma	–/+	Me
5	F/20	IIa	14	12	N	Normal	–/+	T, Me
6	M/21	IIa	9	1	N	Normal	–/+	P, Me
7	F/40	IIIb	19	168	N	Thymoma	–/+	T, P
8	M/65	IIa	8	2	180	Normal	–/+	P
9	M/51	IIa	–	7	90	Normal	–/+	T, P
10	M/57	IVb	20	60	N	Thymoma	–/+	T
11	F/78	IIb	9	14	N	Normal	–/+	P
12	F/71	IIa	–	0.5	N	Normal	–/+	T
13	M/62	IIb	–	12	N	Thymoma	–/+	T
14	F/77	IIb	8	3	N	Normal	–/+	T, Me

*MGFA, Myasthenia Gravis Foundation of America; AChR-Ab, acetylcholine receptor antibody; MuSK-Ab, muscle-specific tyrosine kinase antibody; QMS, Quantitative Myasthenia Gravis Score; PB, pyridostigmine bromide; AD, afterdischarges; F, female; M, male; N, not taken; T, tibial nerve; P, peroneal nerve; Me, median nerve; U, ulnar nerve*.

**Figure 1:**
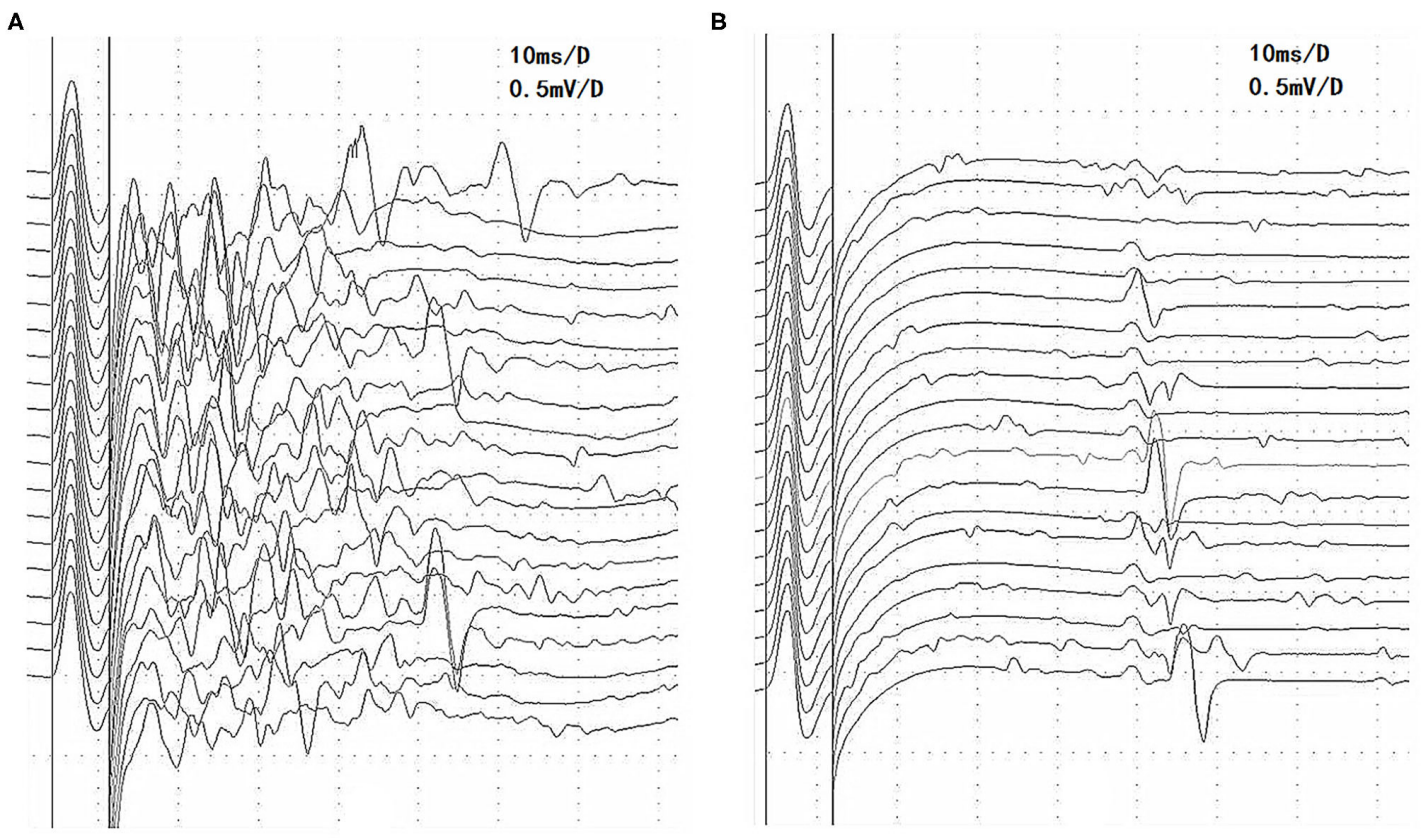
Changes in afterdischarges on the F-wave recording of the tibial nerve before and after pyridostigmine bromide discontinuation in patient 1 with MuSK-MG. **(A)** Afterdischarges appeared before pyridostigmine bromide discontinuation. **(B)** Afterdischarges disappeared after pyridostigmine bromide discontinuation, and the F waves are seen clearly. MuSK-MG, muscle-specific tyrosine kinase antibody-positive myasthenia gravis.

Three of the 14 patients with afterdischarges had MuSK-MG and 11 patients had AchR-MG. Three patients with MuSK-MG had been taking pyridostigmine bromide (400 and 180 mg per day, respectively) regularly prior to EDX examination. These patients continued taking medications prior to the examination because they were concerned that the symptoms would worsen after the withdrawal. Eight of the remaining 11 patients with AchR-MG had never taken AchEIs, 1 patient had not taken an AchEI within 6 months, and 2 patients had been taking pyridostigmine bromide in small doses (90–180 mg per day). After the first EDX examination, pyridostigmine bromide treatment was discontinued in patient 1 because of obvious diarrhea and fasciculation, which were suspected to be muscarinic and nicotinic side effects of the drug. EDX examination was repeated 10 days after drug discontinuation, and the afterdischarges disappeared (Figure 1) (8).

**Figure 2:**
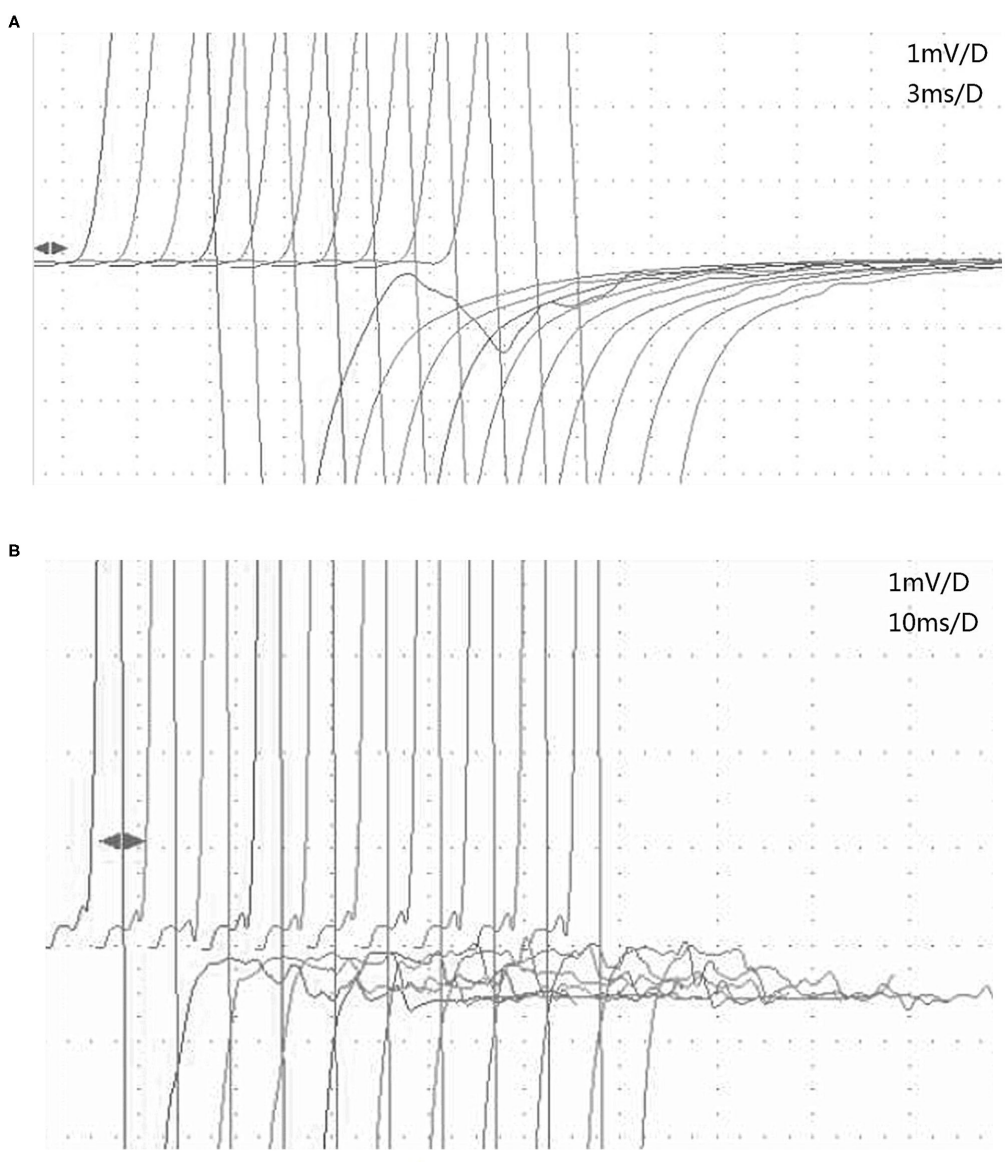
The characteristics of afterdischarges in MuSK-MG and AchR-MG on RNS. **(A)** Afterdischarges appeared on the first stimulation but disappeared on the second 3-Hz RNS of the tibial nerve in patient 2 with MuSK-MG. **(B)** Afterdischarges continued to occur on each 3-Hz RNS of the tibial nerve in patient 6 with AchR-MG. AchR-MG, acetylcholine receptor antibody-positive myasthenia gravis; MuSK-MG, muscle-specific tyrosine kinase antibody-positive myasthenia gravis; RNS, repetitive nerve stimulation.

We discovered an interesting phenomenon by comparing the characteristics of the afterdischarges on RNS in MuSK-MG and AchR-MG. Afterdischarges occurred on the first stimulation, but decreased on the second and subsequent stimulations, which was the same for each frequency (Figure 2A), in 3 patients with MuSK-MG who had been taking pyridostigmine bromide regularly. However, the afterdischarges continued to occur on each stimulation in 4 patients with AchR-MG, and there was no significant amplitude reduction (Figure 2B), which was the same for each frequency.

## Discussion

There were no complaints of muscle twitching, spasm, abnormal sensation, or abnormal sweating in the 14 patients with MG with afterdischarges, except for patients 1 and 2, suggesting that afterdischarges were the early manifestations of PNH. They may even appear before the clinical symptoms (9). The features of the afterdischarges on RNS differed between MuSK-MG and AchR-MG in this study, indicating that the pathological mechanisms underlying the afterdischarges may be different.

### MuSK-MG With Afterdischarges

Excessive doses of AchEI can induce afterdischarges in both MuSK-MG (10) and AchR-MG (11); however, patients with MuSK-MG are more prone to afterdischarges even at therapeutic doses, which has also been confirmed in a mouse model (12).

MuSK not only induces the aggregation of AchR in the postsynaptic membrane, but also draws acetylcholinesterase (AchE) together in the synaptic space (13, 14). MuSK-Abs inhibit the activation of AchE and lead to the accumulation of acetylcholine in the synaptic space (15, 16). The curative effect of AchEI is not satisfactory for MuSK-MG, probably contributing to this pathological mechanism, and muscle weakness is exacerbated in some cases.

Excessive acetylcholine levels in the synaptic space lead to sustained excitement of the postsynaptic membrane during AchEI treatment, which may be the pathogenesis of afterdischarges. This type of afterdischarge originates from the absolute or relative excess of acetylcholine in the synaptic space, hence the term “synaptic afterdischarges” (17). In RNS, the excessive acetylcholine levels gradually became deficient with the progression of the stimulus sequence; thus, the afterdischarges gradually disappeared. Three patients with MuSK-MG in this study had this EDX manifestation. This type of afterdischarge may not persist after AchEI withdrawal, for example, in patient 1.

The nerves of some patients with MuSK-MG with PNH who do not have a history of AChEI medication use rarely generate afterdischarges (18). They may only exhibit muscle twitching or spasm on face or proximal muscles.

The identification of PNH resulting from excessive AchEI levels is helpful for the timely adjustment of drug therapy, which may aid in preventing a cholinergic crisis. A previous study showed that the side effects and afterdischarges induced by pyridostigmine bromide were related to age. The older the patient, the lower the tolerance to pyridinium bromide (11).

### AchR-MG With Afterdischarges

Afterdischarges were also observed in AchR-MG. Patients with AchR-MG were not administered AchEI or only a small dose of pyridostigmine bromide was administered prior to EDX examination in this study, which indicated that the generation of PNH may not be associated with AchEI in these patients. The frequency of thymus abnormalities (including thymic hyperplasia or thymoma) in AchR-MG with afterdischarges accounted for 36% of cases, which is equivalent to 32% of thymus abnormalities in all patients with AchR-MG in this study.

PNH associated with AchR-MG can manifest as Isaacs' syndrome, cramp-fasciculation syndrome, Morvan syndrome, and rippling muscle syndrome (3). This type of immune-induced afterdischarge appears in every RNS, usually with no significant reduction in amplitude, and is thus considered to be a “neural afterdischarge.” Immunosuppressive therapy and plasma exchange are effective treatment modalities. In fact, afterdischarges can also occur in AchR-MG with pyridostigmine bromide overdosage. There was no such case in this group, which may be due to the low dosage of the drug prescribed to the patients in our study (90–180 mg/day).

The autoantibodies associated with PNH and MG sometimes overlap. In PNH, muscular or neural AchR-Abs (19–21) can be detected in addition to voltage-gated potassium-channel antibodies (VGKC-Abs). Similarly, VGKC-Abs can also be detected in MG, besides AchR-Abs or MuSK-Abs (2, 22). This suggests that PNH and MG may be a series of ion channel-related diseases with a common autoimmune mechanism. However, their pathological mechanism remains to be elucidated. We speculated that the presynaptic and postsynaptic membrane may be involved simultaneously or successively in the development of the disease. Myasthenia-related antibodies competitively bind to the receptors on the postsynaptic membrane, affecting the depolarization of the endplate, resulting in MG. Other related antibodies may also bind to the neural AchR on the presynaptic membrane, and cause functional imbalance of the presynaptic membrane, leading to abnormal hyperactivity of nerve endings, which eventually manifests as symptoms of PNH (23, 24). Generally, the clinical symptoms of PNH may be attenuated due to the dystransmission of the neuromuscular junction in MG, while it is more obvious if the myasthenia is alleviated (3). Unfortunately, VGKC-Abs and its related antibodies (e.g., leucine-rich glioma-inactivated protein 1 antibodies and contactin-associated protein-like 2 antibodies) were not found among the patients in the study.

In this study, the characteristics of afterdischarges may reflect their pathophysiological mechanism to a certain extent. A major limitation to this study is its small sample size; thus, future studies with a larger sample size are needed. Another limitation is the lack of patients with AchR-MG with afterdischarges due to AchEI overdosage. Future studies on MG with afterdischarges should ideally include such patients.

## Conclusions

Afterdischarges are the early EDX manifestations of PNH. Afterdischarges can be identified as synaptic or neurogenic, based on their RNS characteristics, which may help guide therapy. Future studies focusing on the pathological mechanism of MG associated with PNH are needed to delve deeper into this condition.

## Data Availability Statement

The original contributions presented in the study are included in the article/Supplementary Material, further inquiries can be directed to the corresponding authors.

## Ethics Statement

The studies involving human participants were reviewed and approved by the Biomedical Research Ethical committee of Shandong Provincial Hospital. Written informed consent for participation was not provided by the participants' legal guardians/next of kin because: The committee waived the need for informed consent as there was no direct contact with the eligible participants or any adverse effects associated with the experimental procedure.

## Author Contributions

LY was responsible for designing research plans, analyzing data, and drafting papers. SG was responsible for collecting and sorting out the data. XC was responsible for final revision. All authors contributed to the article and approved the submitted version.

## Conflict of Interest

The authors declare that the research was conducted in the absence of any commercial or financial relationships that could be construed as a potential conflict of interest.
